# Effect on electrospun fibres by synthesis of high branching polylactic acid

**DOI:** 10.1098/rsos.180134

**Published:** 2018-09-12

**Authors:** Wen Shen, Guanghua Zhang, Xuemei Ge, Yali Li, Guodong Fan

**Affiliations:** 1 School of Food and Biological Engineering, Shaanxi University of Science and Technology, Xi'an 710021, Shaanxi, People's Republic of China; 2 College of Chemistry and Chemical Engineering, Shaanxi University of Science and Technology, Xi'an 710021, Shaanxi, People's Republic of China; 3 College of Life Science, Northwest A&F University, 3 Taicheng Road, Yangling 712100, Shaanxi, People's Republic of China

**Keywords:** high branching PLA, ring opening polymerization, ultra-high molecular weight, hydrophilicity, electrospun, biomaterials

## Abstract

Polylactic electrospun porous fibres have been widely used in tissue engineering scaffolds. However, the application of linear polylactic is limited due to its poor hydrophilicity, which leads to phase separation and has been seldom used in porous fibre preparation. Instead, branching polylactic acts as a new effective method to prepare porous fibres because it can increase polylactic polar property and make it easy to be formulated in the following application. In the current study, we prepared an ultra-high molecular weight of high branching polylactic with glycerol as the initiator by controlling the ring-opening polymerization time, adding amount of catalyst and glycerol. The structure, molecular weight and thermal properties of copolymers were tested subsequently. The result showed that the surface of the high branching polylactic films is smooth, hydrophilic and porous. This branching polylactic formed electrospun porous fibres and possessed a strong adsorption of silver ion. Our study provided a simple and efficient way to synthesize branching polylactic polymer and prepare electrospun porous fibres, which may provide potential applications in the field of biomaterials for tissue engineering or antibacterial dressing compared with the application of linear polylactic and 3-arm polylactic materials.

## 1. Introduction

Polylactic acid (PLA) is a renewable and biodegradable biopolymer, which derives from polysaccharide compounds of natural organic matter such as starch, sugar cane and wheat straw [1]. These polysaccharide compounds could be converted into lactic acid by fermentation and then polymerized to obtain PLA. PLA constructed via chemical reaction such as ring-opening polymerization (ROP) of lactides (i.e. cyclic dimers of lactic acid) [2], direct condensation polymerization or azeotropic dehydration condensation. One advantage of PLA is that this polymer is environment-friendly and can be completely degraded by microorganisms in nature [3,4], and eventually can be converted to carbon dioxide and water. PLA can be used in the food industry [5,6], tissue engineering scaffold [7,8], bacteria production intensification [9], wound dressing [10], moulded parts [11], absorbable suture [12,13], bone-implant [14,15] and drug-delivery system [16,17]. Good tissue affinity is a prerequisite for materials to participate in biological processes [8,18]. While the PLA seems to be a promising material used in many fields such as food chemistry, tissue engineering and biomaterials, the clinical use of PLA is retarded due to its poor hydrophilicity which may result in the low degradation rate of the materials, adhesion of cells and inducement of non-bacterial inflammation [18–20].

Porous PLA electrospun fibres have recently attracted widespread concern in tissue-engineered scaffold [21]. Commonly used electrospinning methods for porous microfibres include phase separation, non-solvent-induced phase separation, inorganic addition, coaxial electrospinning and particle leaching [22,23]. Solvent phase separation was a common method which is based on the formation of different degrees of PLA separation between the solvent and the non-solvent interface. The relationship between the solvent and the non-solvent of PLA in the processing of porous electrospun fibres was investigated in recent studies [23]. However, this method is involved in mixing variety of solvents to make the PLA fibres porous, which makes the electrospinning complex.

The relationship between phase separation and PLA molecular structure is rarely studied in reported studies. In particular, due to different hydrophilicity and crystalline performance, changing the chemical structure of PLA is possible to make porous fibres via a simple solvent method in wet conditions. The molecular morphology of PLA can be divided into two types: linear and branching [24,25]. The linear PLA has poor hydrophilicity and high crystallization ability. On the one hand, the number of intramolecular hydroxyl groups is limited. On the other hand, the linear PLA backbone is difficult to stretch in space, it can form a crystalline hydrophobic structure easily [26]. By lactide ROP, the branching PLA could be synthesized from the centre of polyhydroxy [27]. Lactide ROP is also a common way to obtain high molecular weight PLA [28,29].

**Figure 1.**
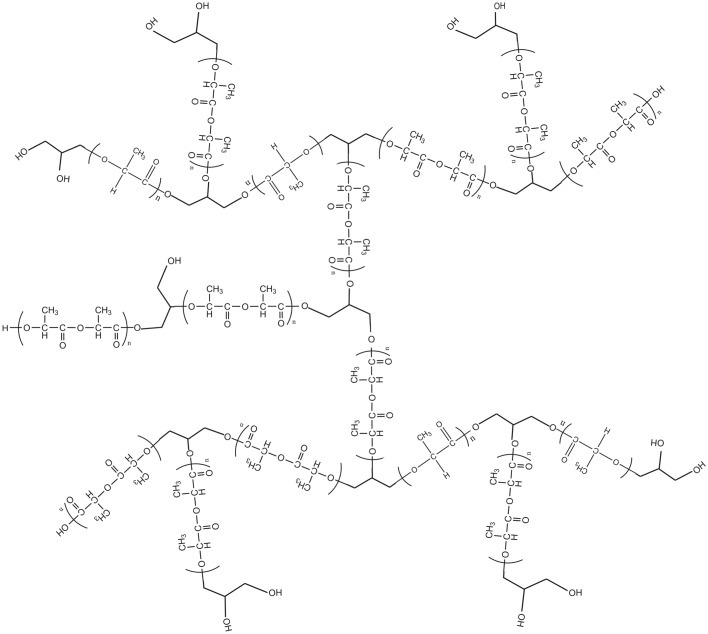
Chemical formula of the highly branched PLA.

To change the hydrophilicity and crystallization of PLA, many approaches have been studied [30,31], including changing the morphology and blending with chemical monomer structure [7,32,33]. In this project, we established a novel condition of high branching PLA preparation. It is generally believed that the copolymerization of glycerol with lactide is an effective way to synthesize 3-arm PLA, but this mechanism may be accompanied by the simultaneous ROP of the lactide and the dehydration of glycerol in a tank and use of metal oxide catalysts [27,29]. This study aims at preparing high branching PLA with hydrophilic ending group by copolymerization of lactide and glycerol. We designed a one-step synthesis process by using toluene as a solvent. The chemical structure of high branching PLA is highlighted and shown in figure 1. The extension of the molecular end hydroxyl enhances the hydrophilicity of PLA. Molecular weight of high branching PLA exceeded the general synthesis methods [34,35], by optimizing the added amount of catalyst [36,37], reaction time [38,39] and the ratio of glycerol to lactide [38]. Also, we characterized the structure and crystallinity of ultra-high molecular weight hydrophilic high branching PLA by IR, ^1^H-NMR, GPC and DSC. The relationship between crystallinity and hydrophilicity of high branching PLA was also discussed in this work. In addition, from the different surface morphology of films characterized by atomic force microscopy (AFM), it is found that high branching PLA has higher density of pore-forming ability, and its porous nanofibres were prepared by electrospinning of phase separation solution. The ability of adsorption of silver ion (Ag^+^) was determined in our study and the results showed that porous nanofibres of the high branching PLA had a high adsorption rate of Ag^+^. The high branching PLA has potential to be used as scaffold for tissue engineering or antibacterial dressing.

## 2. Material and methods

### 2.1. Materials

l-lactide was obtained from Naturework, USA. Glycerol (99%), toluene (98.5%) and other reagents were purchased from Fisher Scientific (Loughborough, UK), all chemicals were used without further purification and all experiments were performed at room temperature if not indicated. The water used in measuring static contact angle (WCA) was of Millipore Milli-Q grade.

### 2.2. Synthesis methods

Stannous octoate (Sn(Oct)_2_) was used as a catalyst (1.5–3.5%, mass fraction), glycerin was used as an initiator, the molar ratio of glycerol to lactide ranged from 1 : 100 to 3 : 100, the initiator and catalyst were added dropwise with a syringe (less than 0.5 ml h^−1^). A predetermined amount of anhydrous toluene was used as a solvent. The reaction was carried out under reflux in a mechanical churning tank under the protection of nitrogen. The mixture was allowed to react at 110°C. A light yellow liquid was obtained and then distillation was repeated until no more toluene came off to get the rough product of high branching PLA. The hot crude was washed with ethanol to remove the catalyst.

Under the same catalytic condition, linear PLA was polymerized by lactide and 3-arm PLA was polymerized by lactide pre-polymer and glycerol. These two types of PLA were used as performance references.

### 2.3. Electrospinning methods

Ten per cent of high branching PLA, linear PLA and 3-arm PLA were dissolved in chloroform, adding 3% ethanol as a pore-forming agent to prepare electrospinning solution. The electrospinning process was carried out in the spinning chamber at a relative humidity of 35% from 20°C to 40°C, voltage was 15 kV, the electric distance was 10 cm, the injection speed was 1.2 ml h^−1^, the syringe needle was no. 6 with a flat end.

Given the situations that other conditions remain unchanged, some electrospinning process for the purpose of producing fibres was carried out at a relative humidity of 45%, with the voltage of 15 kV at 40°C. To further optimize the best condition, electrospinning of fibres was also carried out at a relative humidity of 45%, 40°C with the voltage of 18 kV in our experiment.

### 2.4. The adsorption of Ag^+^ by porous electrospun fibres

According to the method described in §2.3, the fibres prepared by 5 ml electrospun chloroform solution at 40°C were used for this experiment. The porous fibres were added into AgNO_3_ solution at the concentration of 0.5 g/100 ml for 20 min to adsorb Ag^+^ and dried at 30°C.

### 2.5. Characterization of porous electrospun fibres

Fourier transform infrared spectrometry (FTIR) was performed to confirm the chemical structure of the polymer by using FTIR spectrophotometer (FTIR, vector-22, Bruker, Germany). The spectra of all samples were collected by KBr disc method with the scanning range of 500–4500 cm^−1^.

The ^1^H-NMR spectra were recorded by nuclear magnetic resonance instrument (NMR, advance 400 MHz Bruker, Germany). Samples of PLA were dissolved in deuterium generation of chloroform (CDCl_3_).

The thermal properties of PLA were analysed by differential scanning calorimetry (DSC, DSC-204, Netzsch, Germany) under nitrogen flow. The events of interest, i.e. the glass transition temperature (*T*_g_), melting temperature (*T*_m_) were determined from the second heating. Two heating cycles were performed for each sample at a scanning rate of 5°C min^−1^ and the thermograms were recorded between 25°C and 200°C. The first cycle was used to exclude the thermal history of the samples.

The molecular weight and molecular weight distribution (Mw/Mn, PDI) were measured by using gel permeation chromatography apparatus (Viscotek GPCmax, Malvern Instruments Ltd, UK) with RI, viscometer and two-angle light scattering detector from Malvern Instruments. The weight average molecular weight (Mw), the number average molecular weight (Mn) and viscosity average molecular weight (Mp) of the sample were also recorded. The concentration of sample is 10 mg ml^−1^. The mobile phase is tetrahydrofuran (velocity = 1.0 ml min^−1^).

WCA of the sample was measured by using an optical contact angle tester (OCA, DSA100, Kruss, Germany). The linear PLA, 3-arm PLA and high branching PLA were dissolved in CHCl_3_ and evenly coated, and then a dried sample of this film was obtained. Samples were cut at 1 cm width and 3 cm length and 1 μl distilled water was dripped on the surface of the sample and a photo was taken 30 s later. At least, three measurements were performed for each sample.

The same method was used to measure the water contact angle of mats.

Thin films for measuring water contact angles were also used in the AFM (SPA400, Rigaku Corporation, Japan) for observing the surface morphology of the thin films.

The morphology of the fibres was observed by magnifying 1000–5000 times with field scanning electron microscopy (SEM, Hitachi S-4800, Japan).

The amount of silver element on porous fibres was determined by using X-ray photoelectron spectroscopy (XPS, Axis Supra, Kratos, Germany). The binding energy of the Ag^+^ was determined by using the carbon (C 1s: 284.8 eV) as a reference.

## 3. Results and discussions

### 3.1. Characterization of linear PLA, 3-arm PLA and high branching PLA

**Figure 2.**
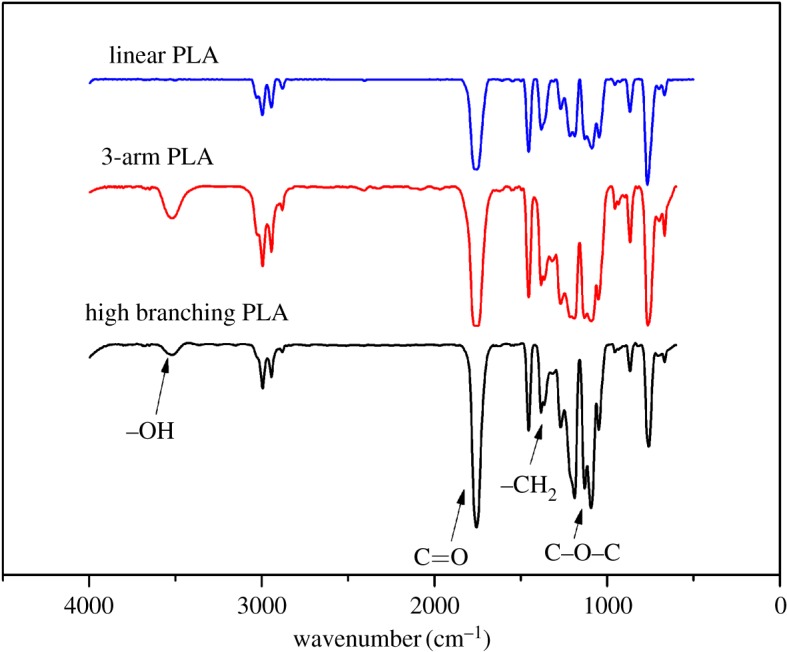
FTIR spectra of linear PLA, 3-arm PLA and high branching PLA.

FTIR spectra of linear PLA, 3-arm PLA and high branching PLA are shown in figure 2. The FTIR spectrum of linear PLA showed the characteristic peak of the 3500 (*ν* O–H), 1743 (*ν* C = O), 1185 (*ν* C–O–C), 952 (*ν* C–C) and 879 (*ν* C–C) cm^−1^. The peak at 3450 (*ν* O–H) cm^−1^ was weaker than those of 3-arm PLA and high branching PLA. In addition, the peak at 1440 cm^−1^ (ν-CH_3_) explained the existence of methyl.

For the FTIR spectra result of 3-arm PLA and high branching PLA, the peak at 1185 (*ν* C–O–C) cm^−1^ had been strengthened. The peaks at 1370 cm^−1^ (ν-CH_3_,-CH_2_) also had been enhanced, which indicates that the ethyl group was detected in the structure. As shown in figure 2, comparing with linear PLA and 3-arm PLA, the superposition of the characteristic peaks in the structure of high branching PLA was the highest, which was also evidence for the formation of new polymers.

**Figure 3.**
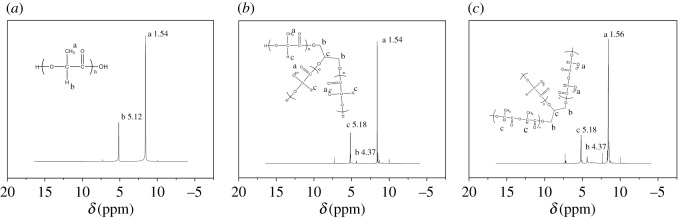
^1^H-NMR spectra of linear PLA (*a*), 3-arm PLA (*b*) and high branching PLA (*c*).

According to the results of ^1^H-NMR, differences of the relative peak intensities were observed in the NMR spectra of the linear PLA (figure 3*a*), 3-arm PLA (figure 3*b*) and high branching PLA (figure 3*c*). As shown in figure 3, the signals at *δ* = 5.12 ppm, and *δ* = 1.54 ppm were assigned to a methine proton and methyl protons of linear PLA block. However, the chemical shifts of the diagnostic peaks appeared to be the same for these three types of PLA polymers. For 3-arm PLA and high branching PLA, the chemical shift at *δ* = 4.37 ppm was assigned to a methylene proton, which indicated that the target polymers were successfully synthesized. In addition, the strength of the peak at 4.37 ppm belongs to high branching PLA which was more remarkable than 3-arm PLA.

Analysis of samples dissolved in tetrahydrofuran mobile phase was performed by using laser detector of GPC, molecular radius of three different types of PLA is listed in table 1. It is found that the linear PLA (Mw = 42 888 ± 37 001 Da) molecular radius was 7.899 ± 2.712 nm, whereas high branching PLA (Mw = 731 607 ± 222 390 Da) molecular radius was 14.832 ± 12.259 nm. While the Mw of 3-arm PLA was 398 210 ± 351 782 Da, its molecular radius was 30.637 ± 22.722 nm. Although the accuracy of laser detector was less than 10 nm, the molecular radius of high branching PLA was closer to the linear PLA. Interestingly, the molecular weight of high branching PLA was more than 10 times that of linear PLA. These results showed that there are obvious differences among various radii of PLA molecular structure. Molecular structure of PLA with different radii increased with the increase of molecular weight, and the high branching PLA molecule possessed a larger nucleus density than that of the 3-arm PLA.

**Table 1.** The molecular radii of three kinds of PLA.

	Mn (Da)	Mw (Da)	Mp (Da)	PDI	Rw (nm)
linear PLA	27 185 ± 25 314	42 888 ± 37 001	34 520 ± 29 219	1.617 ± 0.168	7.899 ± 2.712
3-arm PLA	326 917 ± 299 438	398 210 ± 351 782	617 304 ± 415 225	1.282 ± 0.148	30.637 ± 22.722
highly branching PLA	558 863 ± 153 966	731 607 ± 222 390	577 410 ± 183 556	1.319 ± 0.337	14.832 ± 12.259

**Figure 4.**
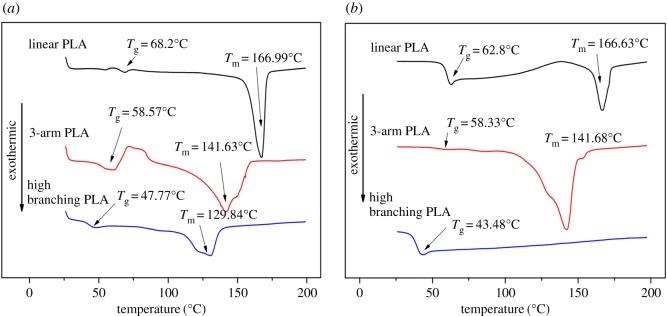
(*a*) Comparison of DSC curves about the first heating scan and (*b*) the second heating scan.

Figure 4 shows typical DSC curves of the linear PLA, 3-arm PLA and high branching PLA. As shown in figure 4*a*, the DSC curve of linear PLA displayed two endothermic peaks at 68.2°C (*T*_g_) and 166.99°C (*T*_m_) from the first heating scan. The DSC curve of high branching PLA had a lower *T*_g_ (47.77°C).

As shown in figure 4*b*, it can be found from the second heating scan that the *T*_g_ and *T*_m_ of linear PLA were 62.8°C and 166.63°C, which were higher than those of 3-arm PLA (58.33°C and 141.68°C) and *T*_g_ (43.48°C) of high branching PLA, respectively, further validating that the branching of PLA chains occurred and hindered the crystallization of PLA. Importantly, no corresponding *T*_m_ can be found on the curve of high branching PLA from the second heating scan. This is because of the knot of high branching PLA chain when it was cooled after the first heating. It also indicates that the degree of its branching was higher than that of 3-arm PLA.

From the second heating scan, the curves of linear PLA displayed an intense cold crystallization peak located approximately in the interval between 130°C and 140°C. As the degree of branching increases, the cold crystallization becomes smaller, which enhanced the difficulty of crystallization. The freedom of high branching PLA chains is larger than those of linear PLA and 3-arm PLA [40].

### 3.2. GPC characterization of synthetic high branching PLA

In this ROP, the reaction was carried out in a tank protected under nitrogen at 110°C. Owing to the thermodegradation and the cleavage of the polymer chains, the attempt at the formation of high branching PLA via the relatively high temperature (greater than 120°C) was not successful.

**Figure 5.**
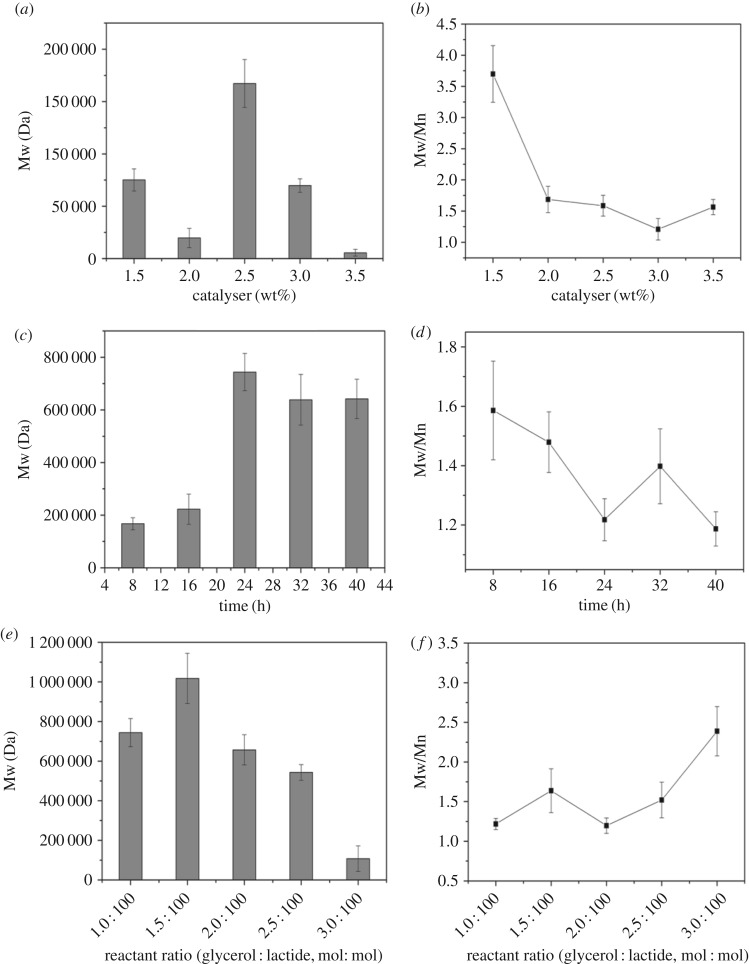
The molecular weight of different catalyst dosage (*a*), polymerization time (*c*) reactant ratio (*e*); the molecular weight distribution of different catalyst dosage (*b*), polymerization time (*d*) reactant ratio (*f*).

The catalytic behaviour of Sn(Oct)_2_ was also examined in this study. When controlling the total mass fraction of Sn(Oct)_2_ in the range of 1.5%–3.5%, 100 : 1 molar ratio of lactide and glycerol were polymerized for 8 h. The changes of Mw and molecular weight dispersion index are shown in figure 5*a,b*, respectively, according to the GPC measurement.

The addition of Sn(Oct)_2_ showed relatively higher activity. Our results indicated that with 1.5% addition of Sn(Oct)_2_, a high branching PLA with Mw over 70 kDa was obtained in the reaction tank and the molecular weight distributes very widely (PDI = 3.7). With increased catalyst dosage, Mw of high branching PLA reached a high level and then decreased. The addition of 2.5% of Sn(Oct)_2_ in the reaction system resulted in a high branching PLA with a higher molecular weight and a broader molecular weight distribution. When the amount of Sn(Oct)_2_ reached 3.5%, Mw of high branching PLA decreased to 5000 Da. That is because the excessive catalyst will create too many catalytic active sites. This activity may lead to a large number of lactide opening rings, which produce more growth centres of polymer, reducing the molecular weight of the product [41]. Although the degree of ROP increased, the degree of dehydration of glycerol is limited, and the product maintained a narrow distribution with low molecular weight. Therefore, the amount of catalyst should be controlled in the reaction system between 2.5% and 3% to obtain high molecular weight of high branching PLA.

Polymers synthesized with 2.5% catalyst at 110°C for 8, 15, 24, 32 and 40 h were analysed to select the best time length of polymerization. The changes of Mw and molecular weight dispersion index were monitored. The data of Mw and PDI versus polymerization time are shown in figure 5*c,d*. As reaction time increased, the molecular weight of high branching PLA showed a gradually increasing trend, with maximum over 700 kDa. The Mw increased with polymerization time from 8 h to 24 h, and PDI decreased linearly from 1.58 to 1.21. Prolonging the reaction time mainly improves the extent of the reaction.

After polymerizing for 24 h, high branching PLA molecular weight reached the maximum. After that, the Mw of the branching polymer no longer increased. Arms of copolymer extension will enable the spatial structure to be closer and increase steric hindrance, which affects the continuation of the reaction. From the characterization of the molecular weight dispersion index, we can observe that the reaction in less than 8 h has a wide molecular weight distribution. PDI tends to reach 1 with this reaction time. Therefore, it can be concluded that molecular weight of high branching PLA tends to be uniform in this reaction system after 24 h, which makes it possible to be used in industrials.

Similar approach has been conducted by using 2.5% Sn(Oct)_2_ as a catalyst and increasing the molar ratio of glycerin/lactide from 1 : 100 to 3 : 100. When the reaction was carried out for 24 h, samples were recovered from different reaction tanks. The changes of Mw and molecular weight dispersion index were determined. As shown in figure 5*e,f*, the Mw of resultant polymers increased at first and then decreased with the increasing addition of glycerin. After the molar ratio of glycerin/lactide above 2 : 100, PDI showed a linearly increasing trend with the ratio. This result showed that the amount of glycerin has a significant influence on polymeric reaction and the polymerization. Glycerol functioned as the best initiate for molecular growth, and Mw reached over 1000 kDa. With the increase of glycerin concentration, polymeric product molecular weight was increasing at first and decreasing later, which was mainly due to the cleavage of the ester bond in the polymer chains. In addition, as the volume of hydroxyl participating polycondensation increased in the system, the product molecular weight is increased. But, when the branching agent exceeds a certain extent, it may create limitations to the polycondensation reaction.

Apparently, increasing the branched initiated agent ratio to 1.5 : 100 in polymerization system and prolonging the reaction time to 24 h can result in a larger molecular weight product. But, PDI increased with the addition of glycerol. In order to obtain higher molecular weights of high branching PLA with larger molecular weight, prolonging the reaction time could be the proper way to achieve this goal. The proper amount of glycerin effectively promoted homogeneity of the reactants, which make the reaction product more equably.

### 3.3. Surface properties of high branching PLA

According to the static WCA test, the hydrophilicity of linear PLA can be increased by changing the molecular structure of PLA.

As shown in table 2, according to the concept of hydrophilic material classification, the WAC of linear PLA was around 120°, which belongs to the hydrophobic material; and the high branching PLA water contact angle was around 80°, which belongs to the hydrophilic material.

**Table 2.** Water contact angles of three kinds of PLA films.

sample	Mw (Da)	*θ* (°)
linear PLA	50 353 ± 12 138	120.09 ± 4.29
3-arm PLA	60 109 ± 14 196	85.96 ± 3.45
high branching PLA	69 699 ± 12 880	79.17 ± 4.15

It is generally accepted that the hydrophilicity of PLA increased with reducing molecular weight [42,43]. According to our experiment, there was a clear correspondence between hydrophilicity and PLA molecular structure [44]. However, the hydrophilic difference between high branching PLA and 3-arm PLA was not obvious. This was because the branching degree of the terminal product highly branched PLA was difficult to control by changing the reaction condition, and the number of the hydroxyls is uncertain.

**Figure 6.**
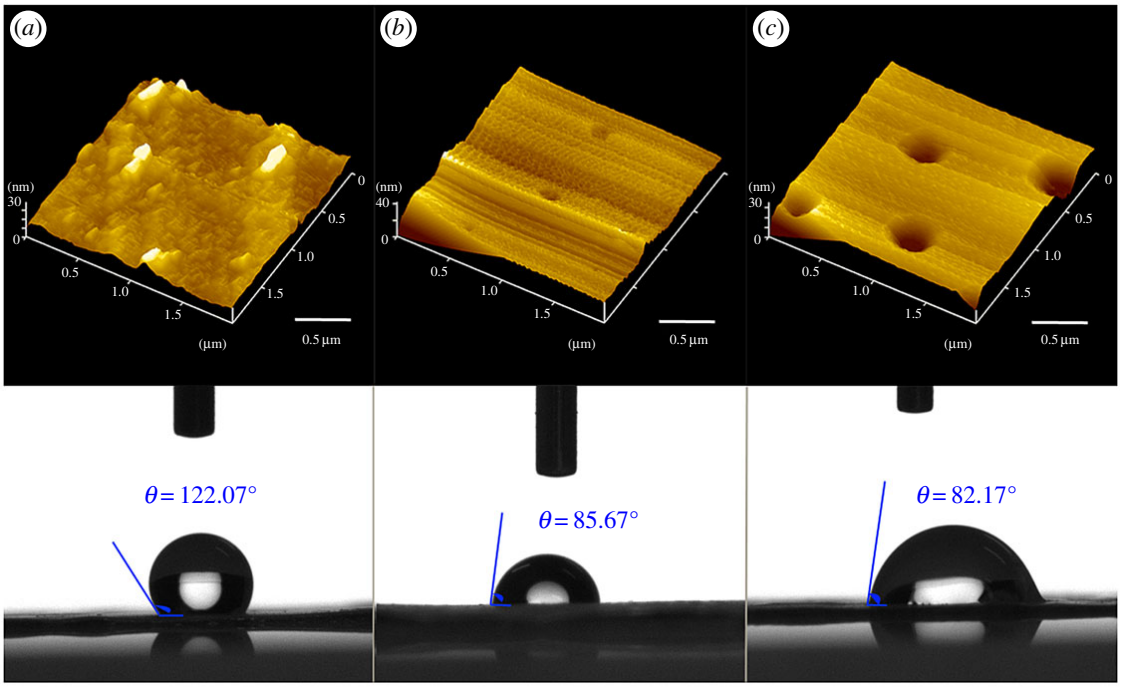
Surface features of linear PLA (*a*), 3-arm PLA (*b*) and high branching PLA(*c*) obtained by AFM and OCA.

The AFM images of linear PLA, 3-arm PLA and high branching PLA film are shown in figure 6. Contact angle was affected by surface topography and roughness [45]. Figure 6*a* shows a sharper peak within the 30 nm range on linear PLA film, indicating that the repulsive force of linear PLA was small and performs a phenomenon of relaxation. In addition, crystallization of linear PLA is more prominent. Obviously, this is a hydrophobic surface structure. In high branching PLA, the hydroxyl groups extend out to space by repulsive interactions. From the analysis of DSC, the degree of intermolecular interlacing is stronger [44]. The surface of the high branching PLA materials was quite smooth (figure 6*c*), which indicates that it has an obvious influence on water contact angle [45]. It shows that the change of molecular structure may lead to an increase in the hydrophilicity of PLA.

It is found that high branching PLA has stronger phase separation ability. Meanwhile, due to the physical characteristic, pores could be formed on the surface of 3-armd PLA and high branching PLA films. We found that on the surface of the high branching PLA, the materials show relatively regular pores with diameter around 500 nm, which results from solvent evaporation. On the surface of 3-arm PLA, as shown in figure 6*b*, the formed pores caused by this phase separation are relatively small in size.

### 3.4. Porous electrospun fibres of high branching PLA

**Figure 7.**
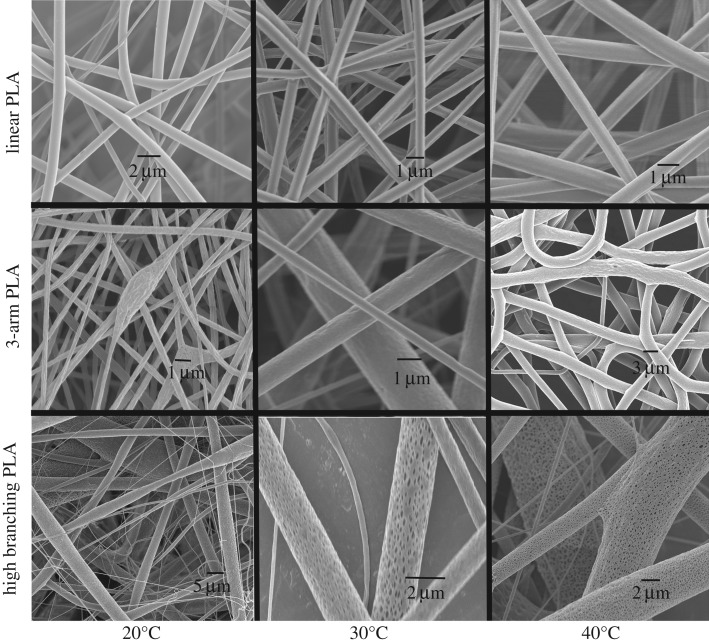
Morphology of electrospun fibres obtained at different temperatures.

As shown in figure 7, high branching PLA showed good pore forming at 20°C, and discontinuous pores appeared on the surface of larger fibres. With the increase of ambient temperature, the number of pores on the surface of high branching PLA fibre increased and its aperture also increased. Linear PLA and 3-arm PLA did not show the same pore-forming ability. The surface of the linear PLA fibres was very smooth at lower electrospinning temperature. However, there was no obvious pore on the linear PLA fibres at 40°C, while 3-arm PLA fibres had pores at 40°C. Both the size and the number of pores on the surface of the fibres formed by linear PLA and 3-arm PLA were smaller and less than that of high branching PLA porous fibres. This shows that high branching PLA still has good pore-forming ability in very weak phase separation solvent. This is consistent with the results of AFM.

**Figure 8.**
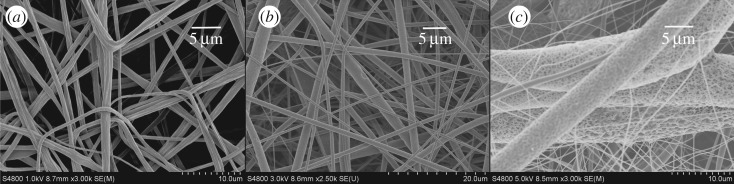
Morphology of linear PLA (*a*), 3-arm PLA (*b*) and high branching PLA (*c*) electrospun fibres obtained at humidity of 45%, 40°C, 15 kV.

In order to gain a better understanding of pore-forming ability of high branching PLA polymer, we adjusted the humidity of electrospinning procedure to 45% and the obtained fibres were observed by SEM as shown in figure 8. There were some grooves formed on the surface of linear PLA fibres. This was probably due to the phase separation which was caused by evaporation of the water and mixed solvent on the surface of the fibres during the stretching processing. Chloroform and water have low solubility, the degree of the water-absorption from the environment was mostly dependent on the nature of jet-flow. So, more phase separations occurred on the surface of 3-arm PLA fibre and high branching PLA fibre. The branching PLA could form much more surface pores because its hydrophilic ability was higher than two other kinds of PLA polymers.

From the results of figures 7 and 8, the electrospun fibre with high branching PLA has a very large diameter distribution characteristic compared with that of the linear PLA, and the diameter distribution of 3-arm PLA fibres is between them. This is due to the high branching PLA jet breaking through the smaller surface tension and forming thick fibres in the electric field, while the branched PLA has a lower *T*_g_, and a finer two-grade jet can be formed on the thicker fibres without crystallization (figure 8*c*).

**Figure 9.**
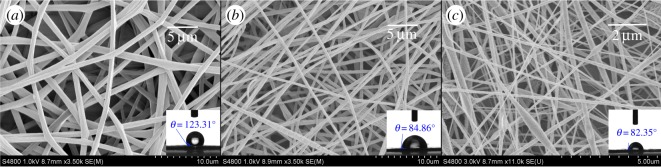
Morphology of linear PLA (*a*), 3-arm PLA (*b*) and high branching PLA (*c*) electrospun fibres obtained at humidity of 45%, 40°C, 18 kV.

As shown in figure 9, we enhanced the stretching strength of jet-flow by increasing the voltage to 18 kV and we normalized the diameter of these three PLA-formed fibres by using this method. It can be concluded that the groove structure was still formed on the surface of the linear PLA fibres and obvious pores formed on the surface of both the 3-arm PLA and high branching PLA polymer-formed fibres. We also determined the contact angle of these three fibres. The results show that linear PLA, 3-arm PLA and high branching PLA-formed fibres have almost the same contact angle with these PLA polymer-formed films (figure 6), indicated that the changing of morphology of electronspun fibres did not affect the hydrophilicity of the materials.

### 3.5. Adsorption of Ag^+^ by porous fibres

**Figure 10.**
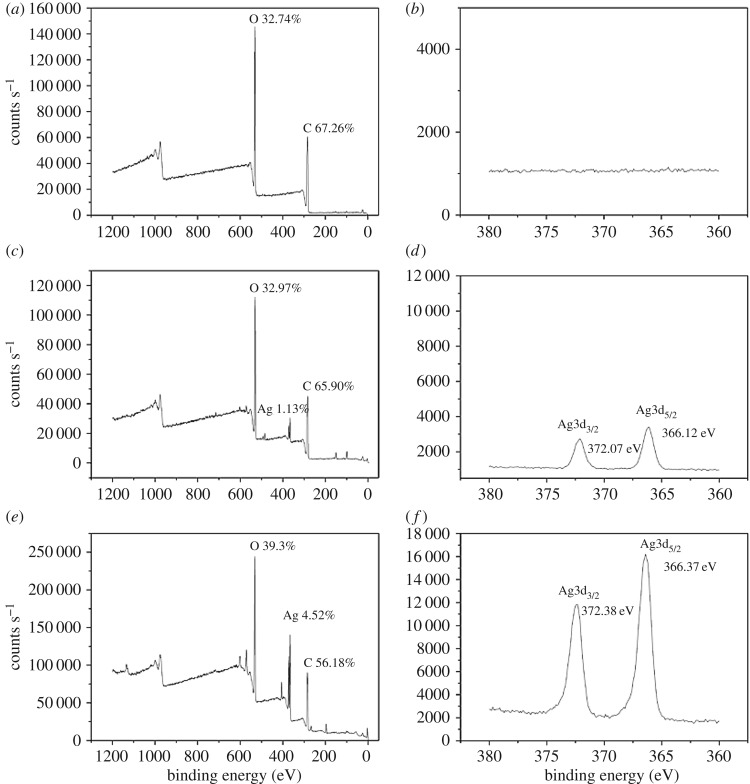
Adsorption of Ag^+^ by different PLA porous fibres (wide spectrum (*a*) and Ag peak (*b*) of linear PLA fibre; wide spectrum (*c*) and Ag peak (*d*) of 3-arm PLA fibre; wide spectrum (*e*) and Ag peak (*f*) of high branching PLA fibre).

Figure 10 illustrates the relationship between adsorption amount of Ag^+^ and different structure of PLA polymer-formed fibres. The XPS characterization of the linear PLA electrospun fibres is shown in figure 10*a,b*, there is nearly no adsorption of silver ions on linear PLA fibres, because this is a kind of non-hydrophilic polymer, and there is no pore on the fibre. However, 3-arm PLA has higher hydrophilicity, whose XPS-wide spectrum and the peaks of Ag^+^ are shown in figure 10*c,d*, respectively. The Ag^+^ on surface of 3-arm PLA fibres occupied 1.13%. The number of pores in high branching PLA fibres is more than that of 3-arm PLA fibres. Figure 10*e,f* shows that the Ag^+^ on surface of high branching PLA fibre occupied 4.52%, although the hydrophilicity is similar (table 2). The Ag3d_5/2_ and Ag3d_3/2_ core-level binding energies for bulk metallic silver lie at 368 and 372 eV, this binding energy indicated that it is not at oxidizing state [46].

The XPS results of the Ag^+^ porous fibres showed that the high branching PLA porous fibres had good adsorption ability of Ag^+^, while linear PLA fibres showed weak adsorption ability of Ag^+^. The Ag^+^ adsorbing ability of 3-arm PLA porous fibres was weaker than high branching PLA porous fibres. The correlation between the amount of Ag^+^ adsorption and the numbers and sizes of the PLA-formed pores was found in our experiments.

The strength of this adsorption capacity is related to the hydrophilic property of three PLA materials [46]. The contribution of Ag^+^ adsorption capacity is not only dependent on porous fibres but also related to hydrophilic PLA materials.

## 4. Conclusion

In this study, we successfully synthesized high branching PLA using a ROP reaction in a tank. The hydrophilicity of this high branching PLA material has been improved significantly compared with that of linear PLA. In the synthesis process, an ultra-high molecular weight (100 kDa) branching PLA with a narrower molecular weight distribution was obtained. The conditions for obtaining the higher molecular weight were as follows: catalyst amount 2.5–3% (wt%), the molar ratio between glycerol and lactide was lower than 2.5 : 100 and the reaction time was longer than 24 h. High branching PLA had smaller molecular volume and complex interlacing structure, whose intermolecular interaction was greater. There was a great difference between the high branching PLA and the linear PLA surface nanostructures which can be observed from the AFM measurement. The results of the static WAC test showed that the WAC of linear PLA was 120°, and WAC of high branching PLA was 80°. Porous electrospun fibres can be prepared under mild conditions by high branching PLA, its porosity was much higher than that of linear PLA and 3-arm PLA fibres. The porous fibres of high branching PLA could achieve the higher ability of absorbing Ag^+^ compared with linear PLA and 3-arm PLA formed fibres.

In conclusion, this high branching PLA had good pore-forming ability, the high porosity electrospun fibre can be obtained under simple conditions, which could be widely used as hydrophilic biomaterials in the fields of tissue-engineered or antibacterial dressing.

## Data accessibility

The analysis data used in this article is linked to a temporary review link: http://dx.doi.org/10.5061/dryad.93tj942 [47].
